# Hand, Foot, and Mouth Disease in Hunan Province, China, 2009-2014: Epidemiology and Death Risk Factors

**DOI:** 10.1371/journal.pone.0167269

**Published:** 2016-11-29

**Authors:** Kai-Wei Luo, Li-Dong Gao, Shi-Xiong Hu, Hong Zhang, Zhi-Hong Deng, Wei Huang, Qian-Lai Sun, Fan Zhang, Si-Yu Zhang, Yu Chen

**Affiliations:** Hunan Provincial Center for Disease Control and Prevention, Hunan, China; University of Florida, UNITED STATES

## Abstract

Hand, foot, and mouth disease (HFMD) is an arising public health problem in Asia, including China. Epidemiological data is necessary to enable judicious public health responses and interventions. We analyzed the epidemiological and laboratory data of 759,301 HFMD cases reported to the Hunan Provincial Center for Disease Control and Prevention from 1 January 2009 to 31 December 2014. Univariate and multivariable conditional logistic regression analyses were used to identify risk factors of fatality in HFMD. The incidence of HFMD was highest among children aged 1–3 years, compared with other age groups. Of the total HFMD cases, 7,222 (0.95%) were considered severe and 338 (0.04%) were fatal. Enterovirus-A71 was the major cause of severe and fatal cases (65.75% and 88.78%, respectively). For severe cases, the median time from symptom onset to diagnosis was 0.5 days (interquartile range [IQR] 0–1.5 days); the median time from diagnosis to severe illness was 2 days (IQR 1–3 days). For fatal cases, the median time from symptom onset to diagnosis was 0.5 days (IQR 0–1.5 days); the median time from diagnosis to death was 1.5 days (IQR 0.5–2.5 days). In multivariable analysis, the abuse of antibiotic, glucocorticoid and pyrazolone in village clinics at basic medical institutions were identified as independent risk factors for HFMD fatal cases. In conclusion, our results suggest that the future direction to control and respond to HFMD is intensive surveillance of enterovirus-A71 and improving the ability to diagnose disease and treat patients, especially in basic medical institutions.

## Introduction

Hand, foot, and mouth disease (HFMD) is an illness of infants and children under 5 years old that is characterized by mouth ulcers and vesicles on the hands, feet, or hips [1]. The infection is usually self-limiting and mild. However, some patients rapidly develop neurological and systemic complications that can be fatal [2]. HFMD is caused by various enteroviruses (EV), most commonly enterovirus 71 (EV-A71) or coxsackie virus A16 (CV-A16) [3–5]. EV-A71 is often associated with major HFMD outbreaks throughout the world [6–9].

In response to the increasing number of HFMD cases in China, especially those that are severe or fatal, the Chinese government established a network-based national surveillance system for HFMD in May 2008. Through this surveillance system, individual cases are reported to the local county- or city-level Center for Disease Control (CDC). The provincial CDC then reviews all case reports, including epidemiological and laboratory data.

In Hunan Province, because of delays in laboratory construction and the network, surveillance data was not reliable until January 2009. To enable the design of better control measures, we conducted the present study of the epidemiological, pathogenic, and clinical characteristics of HFMD in Hunan Province, based on surveillance data collected from January 2009 to December 2014.

## Methods

### Ethics statement

In May 2008, HFMD was added to the list of notifiable diseases in China. According to China’s law on the prevention and treatment of infectious diseases, for the purposes of public health surveillance and response, a diagnosis of a notifiable disease allows the collection of personal information for individual cases. The National Health and Family Planning Commission of China decreed that individual data for all notifiable diseases, including HFMD, is part of an ongoing public health response and thus is exempt from institutional review board assessment. The China CDC and Hunan CDC have strict regulations on the protection of patients’ privacy. The Center for Public Health Surveillance and Information Services (CPHSIS) at the China CDC is responsible for the management of all disease surveillance data. The CPHSIS anonymizes individual HFMD data by deleting personal identifiers, including patient name, parent name, home address, and telephone number. The author were given access to the surveillance data for the purposes of research. The co-authors of this article did not participate in de-identifying the data and do not have the personal identifiers of the HFMD cases.

The authors assert that all procedures contributing to this work comply with the ethical standards of the relevant national and institutional committees on human experimentation, and with the Helsinki Declaration of 1975, as revised in 2008.

### Demographic information of Hunan Province

Hunan Province is located in South Central China, and has a significant HFMD burden. The incidence and mortality rates of HFMD have both ranked among the top 5 diseases in China since 2009 [10].

Hunan Province is divided into 14 prefectures and has an area of 211,800 square kilometers (comparable to the area of Romania) and a population of 71 million. The per capita gross regional product is 33,480 Chinese Yuan (CNY, ~USD $5,444), which is the middle economic level in China [11].

### Surveillance system

Cases of HFMD are reported via a web-based public health surveillance system. Data are sent immediately to the national database. CDC officers at different levels are able to access the information within their jurisdiction as soon as the information becomes available.

From January 2009 to December 2014, the surveillance system included a minimum of 5 mild cases and all severe and fatal cases (defined below) per prefecture per month.

### Diagnostic criteria

A suspected case of HFMD (i.e., clinically diagnosed) was defined as a patient with a papular or vesicular rash on hands, feet, mouth, or hips, with or without fever. A laboratory-confirmed case was defined as a suspected case with laboratory evidence of enterovirus infection (EV-A71, CV-A16, or other EV detected by RT-PCR, real-time PCR or virus isolation) [12, 13].

The cases were classified as mild, severe, or fatal based on the diagnostic criteria established in the Hand, Foot and Mouth Disease Control and Prevention Guide (2009), and in the Hand, Foot and Mouth Disease Treatment Guidelines (2010), published by the Ministry of Health of China [14, 15]. Briefly, the standard diagnosis of a mild case of HFMD includes the presence of a skin rash on the hands, feet, mouth, or hips, with or without fever. A severe case of HFMD was determined according to additional neurological, cardiogenic, or pulmonary symptoms. A fatal case was identified when HFMD was confirmed as the cause of death.

### Pathogen serotype

The CDCs in cities and counties collected and analyzed all case specimens (feces, throat swabs, anal swabs, ulcer fluid, and cerebrospinal fluid) through the standard quality control procedures of the Hunan provincial CDC. In the laboratory, RNA extraction was conducted using a commercial viral nucleic acid extraction kit (Geneaid Biotech, New Taipei City, Taiwan); real-time PCR was performed using a commercially available kit (Bioperfectus, Jiangsu, China), both in accordance with the manufacturer’s instructions. The specifications for specimen collection and real-time RT-PCR were outlined and performed in accordance with the requirements detailed in the chapter on HFMD specimen collection and testing technology in Hand, Foot and Mouth Disease Control and Prevention Guide (2009) [14].

### Data analysis

We included all HFMD cases from 1 January 2009 to 31 December 2014 in the analysis. We determined the rates of case-severity, case-fatality, and case-fatality among severe cases, as follows. The case-severity rate was the number of severe cases divided by the sum of suspected and confirmed cases. The case-fatality rate was the number of deaths divided by the sum of suspected and confirmed cases. The severe case-fatality rate was the number of deaths divided by the sum of severe cases and deaths.

In addition, we analyzed the times from symptom onset to diagnosis, from diagnosis to severity, and from severity to death. In laboratory-confirmed cases, we calculated the proportions of different serotypes.

### Case-control survey investigation

To find the risk factors of fatality in HFMD, 177 fatal cases for which detailed epidemiological data was available were included as the death group in univariate and multivariable conditional logistic regression analyses. As a control group, 2 severe cases were selected for each single fatal case. The questionnaire could be found in S1 Table.

The death and control groups were matched by gender and symptom onset dates (≤14 days apart). The symptom onset date was defined as the date when the patient started to show symptoms of papular or vesicular rash on the hands, feet, mouth, or hips, with or without fever. Univariate conditional logistic regression analyses were used to analyze 22 variables that previous studies had indicated may be associated with fatality in HFMD [16–19]. Those variables that were identified in the present study were then included as candidates in the multivariable conditional logistic regression model, by stepwise selection. SPSS 17.0 software was used to conduct all statistical analyses. A two-sided *P* < 0.05 was considered significant.

## Results

A total of 759 301 cases of HFMD were reported to the surveillance system during the years 2009 to 2014, of which 7,222 (0.9%) were severe cases and 338 (0.1%) were fatal (Table 1). There were 43,111 (5.68%) laboratory-confirmed cases. Of all 759,301 cases, 476,156 (62.7%) were male and 283,145 (37.3%) were female, and the overall male-to-female ratio was 1.68:1. Most of the children affected were cared for at home, but also there were many children affected at kindergartens.

**Table 1:** Case distribution and demographic characteristics of reported HFMD cases in Hunan Province, China, 2009–2014.

		2009	2010	2011	2012	2013	2014	Total
Subjects, n		34,606	112,060	102,311	189,382	108,144	212,798	759,301
Types	Mild	34,516 (99.7)	110,262 (98.4)	102,041 (99.7)	186,250 (98.3)	107,764 (99.6)	210,908 (99.1)	751,741 (99.0)
	Severe	73 (0.2)	1,672 (1.5)	235 (0.2)	3,034 (1.6)	360 (0.3)	1,848 (0.8)	7,222 (0.9)
	Fatal	17 (0.1)	126 (0.1)	35 (0.1)	98 (0.1)	20 (0.1)	42 (0.1)	338 (0.1)
Gender	Male	23,095 (66.7)	71,677 (64.0)	65,626 (64.1)	120,458 (63.6)	66,763 (61.7)	128,537 (60.4)	476,156 (62.7)
	Female	11,511 (33.3)	40,383 (36.0)	36,685 (35.9)	68,924 (36.4)	41,381 (38.3)	84,261 (39.6)	283,145 (37.3)
Child care	Home	25,335 (73.2)	88,461 (78.9)	86,629 (84.7)	165,099 (87.2)	96,093 (88.9)	182,192 (85.6)	643,809 (84.8)
	Kindergarten	7,938 (22.9)	19,119 (17.1)	12,841 (12.5)	18,670 (9.9)	9,776 (9.0)	25,134 (11.8)	93,478 (12.3)
	School	1,165 (3.4)	4,042 (3.6)	2,513 (2.5)	5,176 (2.7)	2,015 (1.9)	4,929 (2.3)	19,840 (2.6)
	Other	168 (0.5)	438 (0.4)	328 (0.3)	437 (0.2)	260 (0.2)	543 (0.3)	2,174 (0.3)
Age y	<1	7,488 (21.6)	27,143 (24.2)	22,805 (22.3)	34,533 (18.2)	27,833 (25.7)	44,887 (21.1)	164,689 (21.7)
	1–3	22,717 (65.6)	68,873 (61.5)	68,091 (66.6)	130,883 (69.1)	70,567 (65.3)	139,419 (65.5)	500,550 (65.9)
	4–5	2,229 (6.4)	7,523 (6.7)	5,827 (5.7)	10,726 (5.7)	4,975 (4.6)	14,646 (6.9)	45,926 (6.1)
	≥6	2,172 (6.3)	8,521 (7.6)	5,588 (5.4)	13,240 (7.0)	4,769 (4.4)	13,846 (6.5)	48,136 (6.3)

The incidence rates of HFMD varied greatly by age, with the greatest number of children aged 6 months to 3 years (Table 2). The incidence rate was very low in infants aged younger than 6 months or children over 6 years old.

**Table 2:** The age-specific incidence rate (per million) in Hunan Province, China, 2009–2014.

Age, y	2009	2010	2011	2012	2013	2014
<0.5	321.36	1504.14	604.8	1805.61	922.56	1504.89
0.5–0.9	10029.45	35801.42	30548.81	44095.71	37091.74	55894.03
1–3	8675.61	26302.56	26003.92	49984.15	26949.50	53244.05
4–5	2772.55	9357.52	7247.95	13341.59	6188.18	18217.51
≥6	35.63	139.78	91.66	217.19	78.23	227.13

**Fig 1:**
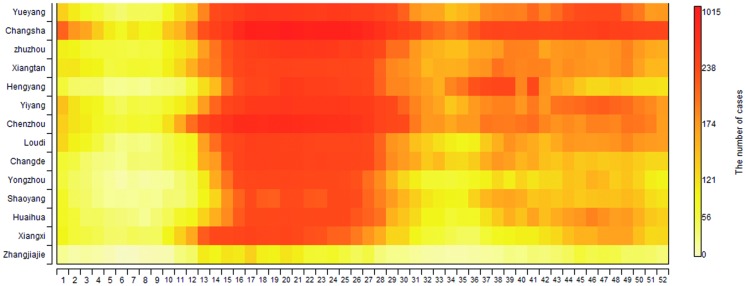
Heat map of weekly case distribution by cities in Hunan Province, 2009–2014. Plotted as the median value of cases in each week of the year from 2009 to 2014. The cities were ordered by longitude from easternmost (top) to westernmost (bottom).

**Fig 2:**
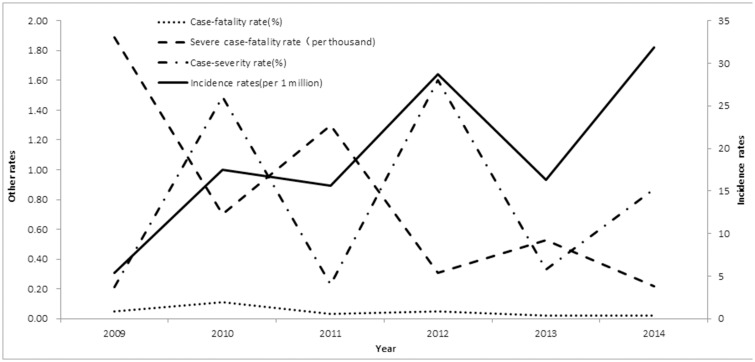
Time trends of incidence and mortality of HFMD, 2009–2014. Incidence rate was the number of HFMD cases divided by population size in each year.

HFMD activity tended to peak semiannually, including a major peak in spring and early summer and then a minor peak in autumn (Fig 1). The major peak from April to July included 53.27% of all reported cases, while the smaller peak between September and November included 14.01%. Notably, these semiannual peaks were more obvious in mid-eastern cities such as Yueyang City than in western cities such as Zhangjiajie City. The number of cases between 2009 and 2014 increased in even-numbered years (i.e., 2010, 2012, and 2014); the average number of cases in even-numbered years was twice that of odd-numbered years. Although the overall incidence of HFMD increased during the 6 years (from 5.42 million to 31.83 million) the fatality rate dropped from 0.05% to 0.02%, and the severe case-fatality rate dropped from 1.89% to 0.22% (Fig 2).

**Fig 3:**
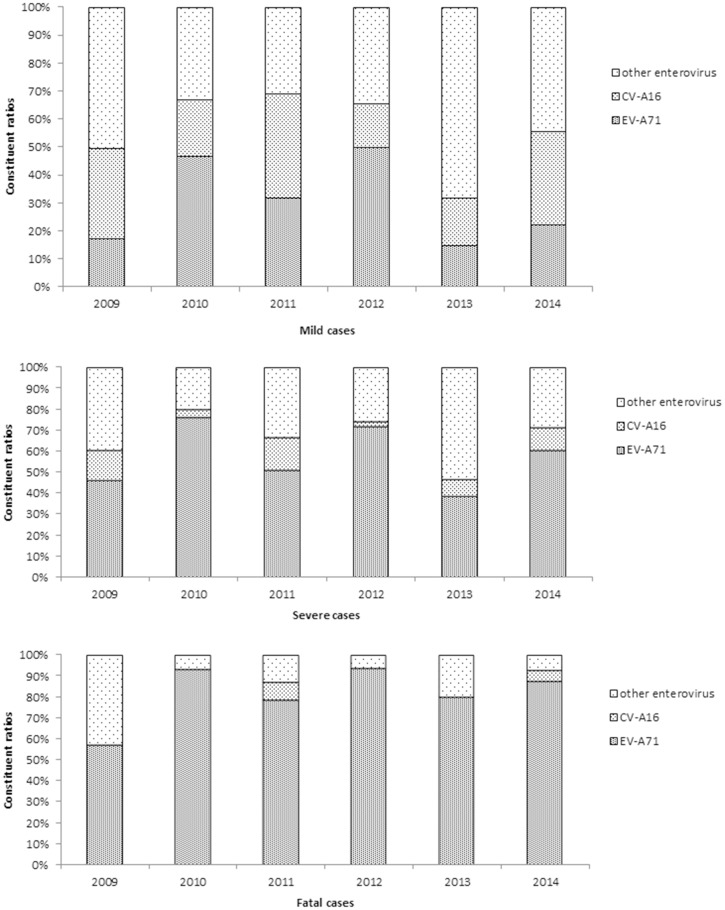
Constituent ratios of enterovirus serotypes in laboratory-confirmed cases of HFMD by clinical severity in Hunan Province, 2009–2014.

A total of 43,111 cases were tested in the laboratory, 36,007 mild, 6,871 severe, and 233 fatal cases, respectively. Among the laboratory-tested cases, 62.59% (26 985 cases) were confirmed HFMD. Enteroviruses (e.g., EV-A71, CV-A16, and untyped enteroviruses) were identified as the cause in ~75% of the mild cases. In particular, in mild cases untyped enteroviruses were the cause in 43.35% and EV-A71 were the cause in 31.85%. On the other hand, EV-A71 was the major cause in 65.75% and 88.78% of severe and fatal cases, respectively (Fig 3). Notably, EV-A71 predominated in 2010 and 2012 when the most serious epidemics were recorded. However, when other enteroviruses became more dominant than EV-A71 in 2009, 2013, and 2014, the case-severity rates were much lower compared to other years.

**Fig 4:**
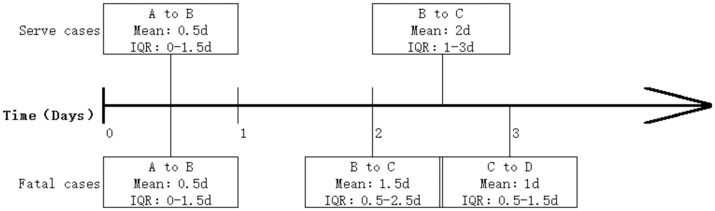
Time interval of HFMD development in severe cases and fatal cases. (A) A:Symptom onset; B: diagnosis; C: start of severe illness; D: death. (B) The time 0 is symptom onset, and the mean in each block is in reference to the location of the previous block.

The median time of severe cases from symptom onset to diagnosis was 0.5 days (interquartile range [IQR] 0–1.5 days), while the median time from diagnosis to disease severity was 2 days (IQR 1–3 days; Fig 4). For fatal cases, the median time from symptom onset to diagnosis was the same as that of severe cases, but the time from diagnosis to severe illness was 1.5 days (IQR 0.5–2.5 days).

The univariate conditional logistic regression analysis showed no significant difference between the death and control (severe) groups with regard to the following: age; occupation; birth order of patient; gestational weeks of the mother; delivery mode; birth weight; pattern of infant feeding; style of child-care; educational level of the people caring for the children; or histories of obstetric labor complications, congenital malformations, congenital heart disease, or drug or food allergies. On the other hand, eight variables were significantly associated with fatal cases: reside in city areas, first visit in higher level hospitals, correct diagnosis when first visit, shorter time between symptom onset to first visit, shorter time between symptom onset to county hospital were protective factors, while using antibiotic in village clinics, using glucocorticoid in village clinic, using pyrazolone in therapeutic process were risk factors(Table 3). Subsequently these eight variables were entered in multivariable conditional logistic regressions. All factors remained significantly associated with fatal cases except for correct diagnosis when first visit. (Table 4).

**Table 3:** Distribution of case and control groups and univariate conditional logistic regression model.

		Death group	Control group	OR (95% CI)	*P*
Subjects, n		177	354		
Age, y	<1	21 (11.9%)	13 (3.7%)	2.019 (0.457–8.920)	0.354
	1–3	146 (82.5%)	320 (90.4%)	0.570 (0.151–2.155)	0.408
	4–5	6 (3.4%)	16 (4.5%)	0.469 (0.093–2.357)	0.358
	≥6	4 (2.3%)	5 (1.4%)	Reference	—
Profession type	Isolated children	159 (89.8%)	298 (84.2%)	1.067 (0.263–4.324)	0.927
	Childcare	15 (8.5%)	50 (14.1%)	0.600 (0.134–2.692)	0.505
	Student	3 (1.7%)	6 (1.7%)	Reference	—
Residence	Rural	140 (79.1)	227 (64.1%)	3.152 (1.824–5.449)	<0.0001
	Rural-urban fringe	19 (10.7%)	35 (9.9%)	2.775 (1.307–5.892)	0.008
	Urban	18 (10.2%)	92 (26.0%)	Reference	—
First-visit hospital level	Village	88 (49.7%)	74 (20.9%)	5.203 (2.785–9.719)	<0.0001
	Township	40 (22.6)	113 (31.9%)	1.549 (0.807–2.972)	0.189
	County	33 (18.6%)	97 (27.4%)	1.488 (0.760–2.913)	0.246
	City	16 (9.0%)	70 (19.8%)	Reference	—
Correct diagnosis upon first visit	Y	44 (24.9%)	149 (42.1%)	2.197 (1.471–3.280)	<0.0001
	N	133 (75.1)	205 (57.9%)	Reference	—
Time from onset to first visit, d	0	24 (13.6%)	102 (28.8%)	0.118 (0.067–0.206)	
	1–2	55 (31.1%)	203 (57.3%)	0.135 (0.086–0.213)	<0.0001
	≥3	98 (55.4%)	49 (13.8%)	Reference	—
^a^ Time from onset to county hospital, d	0	25 (16.23%)	96 (28.74%)	0.187 (0.109–0.319)	<0.0001
	1–2	30 (19.48%)	167 (50.00%)	0.129 (0.079–0.211)	<0.0001
	≥3	99 (64.29%)	71 (21.26%)	Reference	—
Antibiotic given at village clinic	Y	64 (36.2%)	58 (16.4%)	2.890 (1.906–4.382)	<0.0001
	N	113 (63.8%)	296 (83.6%)	Reference	—
Glucocorticoid given at village clinic	Y	11 (6.2%)	4 (1.1%)	5.798 (1.819–18.481)	0.003
	N	166 (93.8%)	350 (98.9%)	Reference	—
Pyrazolone during therapy	Y	50 (28.2%)	25 (7.1%)	5.181 (3.074–8.732)	<0.0001
	N	127 (71.8%)	329 (92.9%)	Reference	—

^a^ Some cases did not went the county hospitals.

**Table 4:** Adjusted odds ratio (OR) and 95% confidence interval (CI) derived from multiple conditional logistic regression model.

		OR (95% CI)	*P*
Residence			0.015
	Rural	3.205 (1.445–7.112)	0.004
	Rural-urban fringe	3.313 (1.090–10.071)	0.035
	Urban	Reference	—
First-visit hospital level			<0.0001
	Village	18.798 (7.880–44.844)	<0.0001
	Township	5.129 (2.102–12.515)	<0.0001
	County	2.974 (1.164–7.596)	0.023
	City	Reference	—
Time from onset to first visit, d			<0.0001
	0	0.030 (0.011–0.077)	<0.0001
	1–2	0.141 (0.069–0.288)	<0.0001
	≥3	Reference	—
^a^ Time from onset to county hospital, d			<0.0001
	0	0.032 (0.012–0.080)	<0.0001
	1–2	0.067 (0.032–0.142)	<0.0001
	≥3	Reference	—
Antibiotic given at village clinic	Y	21.116 (9.384–47.518)	<0.0001
	N	Reference	—
Glucocorticoid given at village clinic	Y	6.641 (1.087–40.565)	0.04
	N	Reference	—
Pyrazolone during therapy	Y	3.046 (1.202–7.715)	0.019
	N	Reference	—

^a^ Some cases did not went the county hospitals.

## Discussion

In this study, we investigated more than 750,000 cases of HFMD reported from the national enhanced surveillance system from 2009 to 2014. This report provides the first comprehensive understanding of the epidemiological burden of HFMD in Hunan Province, a disease that causes illness and death among children less than 5 years old [10, 20–22]. Hunan Province lies in the middle of China with an economy that is mid-level. The province also ranks among the first for both incidence and mortality rates of HFMD. As in many other provinces in China, the incidence rate of HFMD infections increased from 2009 to 2014, reflecting both the rapid increase of HFMD in China and the improvement in HFMD case reporting and surveillance [23, 24]. However, despite the increase in incidence the case-fatality rate dropped steadily, which reflects improvement in the treatment of severe cases in recent years.

As in other southern provinces in China, HFMD in Hunan had semiannual peaks of activity, including a major peak in spring and summer, and a smaller peak in autumn [25]. However, this pattern changed by latitude, and only one peak in summer was detected in provinces in the north [26]. A reasonable explanation of this phenomenon could be the influence of temperature and humidity on the activity of enteroviruses. The high incidence in every other year was likely a result in periodic depletion and replenishment of the susceptible population. Climate and geographic factors may influence the periodicity of incidence as well.

The age profile of HFMD was consistent with reports from other provinces in China and other countries [27, 28]. The young-age characteristic of HFMD infection suggests that enteroviruses are highly transmissible, while the relatively low incidence rate in infants younger than 6 months was most likely due to protection by maternal antibodies. The high incidence rate in children aged 0.5–3 years (mostly children living at home) and 4–5 years old (mostly children in kindergartens), suggests that these populations are key to preventing and controlling HFMD.

We also found that more boys were affected by HFMD than girls, and the gender distribution was similar to that observed in other Asian regions [29, 30]. This may indicate that boys are more susceptible to enterovirus infection. It is worth noting that the male-to-female ratio declined from 2.01 in 2009 to 1.53 in 2014. This trend needs further monitoring.

The data derived from surveillance and laboratory monitoring in Hunan showed that HFMD morbidity and mortality were associated with infections by EV-A71, CV-A16, and untyped enteroviruses. In particular, untyped enterovirus was the predominant cause of mild laboratory-confirmed cases, but EV-A71 predominated in severe and fatal cases. This may be because EV-A71 usually invades the nervous and respiratory systems and leads to acute neurologic and respiratory diseases such as paralysis, encephalitis, aseptic meningitis, and pneumonedema [31]. HFMD EV-A71-associated neurological syndrome has increased in many countries during the past 5 years, especially in the Asia-Pacific region [32, 33]. In general, since the predominant virus serotype varies each year, the periodicity of HFMD may be complicated by interference between causative enterovirus serotypes [32, 33]. A limitation of our study is that we did not test other enterovirus serotypes, such as CV-A6 or CV-A10, which are becoming more predominant in some provinces of southern China [34].

Fatalities due to HFMD are a huge burden to patients’ families and society, and doctors and the public health department want to know why some severe cases recover but others do not. Thus, we investigated risk factors that may differentiate clinical outcomes in severe cases, enable doctors to monitor and treat accordingly, and guide prevention and control measures by public health departments. The results showed that inappropriate utilization of glucocorticoids, pyrazolones, and antibiotics in village clinics were the risk factors of fatality—the outcomes of patients given these medications differed drastically from the control group.

Glucocorticoids, such as dexamethasone, are commonly used by rural practitioners in China to treat fever, and it is appropriate to use glucocorticoid to diminish inflammation in severe cases of HFMD. However, early use of glucocorticoid in mild cases caused by human EV-A71 has been found to worsen the disease [35,36]. Also, using antibiotics in village clinics was identified as a risk factor of HFMD fatality. A possible reason is that the combination of antibiotics, glucocorticoids, vitamins, and fluid therapy to treat fever is habitually abused in grassroots medical institutions in some villages of China. Thus, we suggest that antibiotics and glucocorticoids should be used with caution in grassroots medical institutions, especially in the early stages of illness.

In the present study, another risk factor of fatality was treatment with pyrazolone; this result is similar to studies reported previously [36, 37]. Pyrazolone derivatives (including aminopyrine, analginum, and phenylbutazone) have been banned and restricted in many developed countries for various adverse drug reactions [38]. However, pyrazolone derivatives are still used broadly in Chinese rural areas for its low price and powerful antipyretic effects. Therefore, we encourage the Hygiene Administrative Department to restrict the use of pyrazolone drugs, especially for children. Most rural patients first seek medical care in the village, followed by township, county, and then city hospitals. However, only hospitals at the county level and higher admit HFMD as severe cases, in accordance with national rules. Therefore, improvement of HFMD awareness and diagnostic measures at lower level hospitals is crucially important to rescue severe cases.

In conclusion, HFMD is an increasingly important public health problem in Hunan Province of China. EV-A71 infection results in severe and fatal cases. Preschool children are at highest risk of HFMD infection. It is important to avoid the injudicious use of drugs, especially antibiotics, in basic medical institutions. Taken together, future efforts to control and respond to HFMD should focus on intensive surveillance of EV-A71, improving the ability to diagnose and treat illness, especially in basic medical institutions.

## Supporting Information

S1 Table The severe or death hand, foot, and mouth disease case questionnaire. (DOC)
