# Supplementary material for: Hand, Foot, and Mouth Disease in Hunan Province, China, 2009-2014: Epidemiology and Death Risk Factors
Source: PLoS One. 2016 Nov 29;11(11):e0167269. doi: 10.1371/journal.pone.0167269 (PMC5127556; doi:10.1371/journal.pone.0167269)
Supplement: S1 Table — (DOC) [file pone.0167269.s001.doc]

# The severe or death hand, foot, and mouth disease case questionnaire

Case classification: severe disease death

Case number:

1. **Basic information of children patients and their families**
2. Gender: 1male 2female
3. Age: year(s) month(s)
4. Date of birth: (month/day/year) whether lunar birthday: 0 no 1 yes
5. Height: cm weight: kg
6. Classification: scattered children kindergarten children student other
7. Current residence time: year(s) month(s)
8. Account type: permanent residents(local account or current residence time > 6 months) floating population(current residence time < 6 months)
9. Current residence type: rural area fringe 3urban area
10. **The situation of falling ill, visiting hospital and treatment**

B1 1.Date of onset: (month/day/year)

B2 2.First date of visit: (month/day/year)

B21 Initial treatment name of hospital:

B22 Initial treatment hospital type: village(private) clinics villages and towns(community) hospital county hospital municipal level and above hospital

B23 Whether diagnosed HFMD in initial diagnosis: 0 no 1 yes

B3 3.Diagnosis of severe dates: (month/day/year)

B31 Diagnosis of severe hospital name:

B32 Diagnosis of severe hospital type: village(private) clinics villages and towns(community) hospital county hospital municipal level and above hospital

B4 4.Whether visit village(private) medical institutions: 0 no 1 yes, date of visiting:b41 (month/day/year)

B42 Treatment time: day(s)

B43 Whether diagnosed HFMD: 0 no 1 yes

B44 Whether drug treatment: 0 no 1 yes 2 unknown

B451 Route of administration: oral 0 no 1 yes, intramuscular injection 0 no 1 yes, intravenousb453 0 no 1 yes, b454 rectal administration 0 no 1 yes, otherb455 0 no 1 yes

Whether use antipyretic drugs: 0 no 1 yes 2 unknown, use date: (month/day/year)

Drug specific name :

Whether use steroid hormone drugs like Dexamethasone: b4b1 b4b2 0 no 1 yes 2 unknown, use date:b4b2 (month/day/year)

Drug specific nameb4b3:

Whether use antibiotics:b4c1 b4c2 0 no 1 yes 2 unknown, use date:b4c2 (month/day/year)

Drug specific nameb4c3:

Whether use antiviral drugs:b4d1 b4d2 0 no 1 yes 2 unknown, use date:b4d2 (month/day/year)

Drug specific nameb4d3:

B5 5.whether visit villages and towns(community) institutions:b5 b51 0 no 1 yes, date of visiting:b51 (month/day/year)

B52 treatment time:b52 b53 day(s) whether diagnosed HFMD: b53 0 no 1 yes

B54 whether drug treatment:b54 0 no 1 yes 2 unknown

B551 route of administration: oralb551 0 no 1 yes, b552 intramuscular injectionb552 0 no 1 yes, b553 intravenousb553 0 no 1 yes, b554 rectal administrationb554 0 no 1 yes, b555 otherb555 0 no 1 yes

Whether use antipyretic drugs:b5a1 b5a2 0 no 1 yes 2 unknown, use date: b5a2 (month/day/year)

Drug specific nameb5a3:

Whether use steroid hormone drugs like Dexamethasone: b5b1 b5b2 0 no 1 yes 2 unknown, use date:b5b2 (month/day/year)

Drug specific nameb5b3:

Whether use antibiotics:b5c1 b5c2 0 no 1 yes 2 unknown, use date:b5c2 (month/day/year)

Drug specific nameb5c3:

Whether use antiviral drugs:b5d1 b5d2 0 no 1 yes 2 unknown, use date:b5d2 (month/day/year)

Drug specific nameb5d3:

Other drugs:b56

B6 6.whether visit county institutions: b6 b61 0 no 1 yes, date of visiting:b61 (month/day/year)

B62 treatment time:b62 b63 day(s) whether diagnosed HFMD: b63 0 no 1 yes

B64 whether drug treatment:b64 0 no 1 yes 2 unknown

B651 route of administration: oralb651 0 no 1 yes, b652 intramuscular injectionb652 0 no 1 yes, b653 intravenousb653 0 no 1 yes, b654 rectal administrationb654 0 no 1 yes, b655 otherb655 0 no 1 yes

Whether use antipyretic drugs:b6a1 b6a2 0 no 1 yes 2 unknown, use date: b6a2 (month/day/year)

Drug specific nameb6a3:

Whether use steroid hormone drugs like Dexamethasone: b6b1 b6b2 0 no 1 yes 2 unknown, use date:b6b2 (month/day/year)

Drug specific nameb6b3:

Whether use antibiotics:b6c1 b6c2 0 no 1 yes 2 unknown, use date:b6c2 (month/day/year)

Drug specific nameb6c3:

Whether use antiviral drugs:b6d1 b6d2 0 no 1 yes 2 unknown, use date:b6d2 (month/day/year)

Drug specific nameb6d3:

Other drugs:b66

B7 7. Whether visit municipal level and above institutions: b7 b71 0 no 1 yes, date of visiting:b71 (month/day/year)

B72 treatment time:b72 b73 day(s) whether diagnosed HFMD: b73 0 no 1 yes

B74 whether drug treatment:b74 0 no 1 yes 2 unknown

B751 route of administration: oralb751 0 no 1 yes, b752 intramuscular injectionb752 0 no 1 yes, b753 intravenousb753 0 no 1 yes, b754 rectal administrationb754 0 no 1 yes, b755 otherb755 0 no 1 yes

Whether use antipyretic drugs:b6a1 b7a2 0 no 1 yes 2 unknown, use date: b7a2 (month/day/year)

Drug specific nameb7a3:

Whether use steroid hormone drugs like Dexamethasone: b7b1 b7b2 0 no 1 yes 2 unknown, use date:b7b2 (month/day/year)

Drug specific nameb7b3:

Whether use antibiotics:b7c1 b7c2 0 no 1 yes 2 unknown, use date:b7c2 (month/day/year)

Drug specific nameb7c3:

Whether use antiviral drugs:b7d1 b7d2 0 no 1 yes 2 unknown, use date:b7d2 (month/day/year)

Drug specific nameb7d3:

Other drugs:b76

B8 8.the last date of hospitalization:b8 (month/day/year)

B81 the type of hospital:b81 village(private) clinics villages and towns(community) hospital county hospital municipal level and above hospital

B82 Admission condition:b82 critical severe mild

B83 Exacerbations date after discharge:b83 (month/day/year)

B84 whether into the ICU ward:b84 b85 0 no 1 yes into the ICU ward date:b85 (month/day/year) the ICU ward time:b86 day(s)(if less than one day, convert to decimal)

B87 whether endotracheal intubation(mechanical ventilation):b87 b88 0 no 1 yes, date to start endotracheal intubation(mechanical ventilation):b88 (month/day/year) endotracheal intubation(mechanical ventilation) time:b89 day(s) (if less than one day, convert to decimal)

B8d death date:b8d (month/day/year)

Death diagnosis: the main diagnosisb8z:

Other diagnosisb8zq:

1. **Past medical history and other relevant information**

1.birth weight:c1 c1a (g) gestational age: c1a c1b (if gestational age is unknown, answer whether premature birth c1b 0 no 1 yes)

Parity: c1c parity c1d c1e delivery times mode of delivery:c1e cesarean section natural childbirth

C2 2.whether childbirth complications:c2 0 no 1 yes (if yes, please indicate it c2a )

C3 3.feeding mode: c3 breast milk mixing milk powder other

C4 4.whether suffer from congenital disease like congenital heart disease, congenital malformations, etc.:c4 c4a 0 no 1 yes, if yes, name of disease: c4a

C5 5.whether suffer from immune deficiency diseases: c5 c5a 0 no 1 yes, if yes, name of disease: c5a

C6 6.whether have drug or food allergies: c6 c6a 0 no 1 yes, if yes, name of drug or food: c6a

C7 7.whether have vaccination card:c7 0 no 1 yes

8. A month before the onset, whether get a vaccination(if without vaccination card, ask the parents):c8 0 no 1 yes 9 unknown

C81n vaccine name c81 c81mdy date of vaccination:c81mdy (month/day/year)

C82n vaccine name c82 c82mdy date of vaccination:c82mdy (month/day/year)

C81n vaccine name c83 c83mdy date of vaccination:c83mdy (month/day/year)

C9 9. A month before the onset, whether suffer from Infectious Disease like measles, chicken pox, influenza, cold, rubella, Mumps, etc.: c9 0 no 1 yes

C10 date of onset:c10 c10a (month/day/year) name of disease:c10a

C11 11.3 month before the onset, whether have a fever:c11 0 no 1 yes

C12 12.Whether use antipyretic drugs: c12 0 no 1 yes

C13a 13.Whether use drugs like Aminopyrine c13a 0 no 1 yes c13b Analginc13b 0 no 1 yes c13c Antondine c13c 0 no 1 yes c13d lysisin aspirin c13d 0 no 1yes c13e steroid hormone drugs (Dexamethasone, etc) c13e 0 no 1 yes

C14 14.Who mainly look after the children at home: c14 parents grandparents relatives baby-sitter

C14a caretakers’s education time: c14a year(s)

C14b caretakers’s education level: c14b illiteracy elementary junior high school senior high school/technical secondary school college or above level

C15 15. Whether children under 14 years of age who always play with the child before onset get onset: c15 0 no 1 yes number of cases: c15a person(s)

C16 16. During 3-7 days before onset, whether visit hospital for other disease: c16 0 no 1 yes date of visiting hospital :c16a (month/day/year) c16b department:c16b c16c reason for visiting: c16c

1. **Specimen collection and test results**

D1 1.Whether collect specimen: d1 0 no 1 yes

D2 Test results: EV71 positive d3a d3b CoxA16 positive d3b d3c other Enteroviruses positive d3c d3d negative d3d 0 no 1 yes

1. **The clinical symptoms and signs**

E1 1.whether have a fever: e1 e11 0 no 1 yes fever start date:e11 e12(month/day/year) duration of fever:e12 day(s)

E2 2. measuring body temperature at first time: e2 e21℃ highest temperature before treatment:e21 e22℃ highest temperature after treatment:e22 ℃

E3 3.whether the onset of rashes: e3 e31 0 no 1 yes date of the onset of rashes :e31 e32(month/day/year) duration of rashes:e32 day(s)

E33a rashes type: macula e33a e33b 0 no 1 yes pimples e33b e33c 0 no 1 yes herpes e33c e33d 0 no 1 yes other e33d 0 no 1 yes

E34a rash site: hands e34a e34b 0 no 1 yes feet e34b e34c 0 no 1 yes mouse e34c e34d 0 no 1 yes buttock e34d 0 no 1 yes e34e four limbs e34e e34f 0 no 1 yes trunk e34f e34g 0 no 1 yes other e34g 0 no 1 yes

E35a at which site herpes or ulcer locate in the mouth: cheek e35a e35b pharynx isthmus e35b e35c 0 no 1 yes other e35c 0 no 1 yes

E4 4.whether cough: e4 0 no 1 yes

E5 5.other symptoms: e5

**If the following symptoms have already happened when “before admission” or “admission”, fill in “1” meaning yes, or else fill in “0” meaning no. If the following symptoms happened when “after admission”, fill in the date when those happened first time.**

**Nervous system**

aa1 headache before admission aa1 ab1 admission ab1 ac1 after admission ac1

aa2 poor spirit before admission aa2 ab2 admission ab2 ac2 after admission ac2

aa3 easily frightened before admission aa3 ab3 admission ab3 ac3 after admission ac3

aa4 dysphoria before admission aa4 ab4 admission ab4 ac4 after admission ac4

aa5 convulsion before admission aa5 ab5 admission ab5 ac5 after admission ac5

aa6 frequent twitch before admission aa6 ab6 admission ab6 ac6 after admission ac6

aa7 convulsions before admission aa7 ab7 admission ab7 ac7after admission ac7

aa8 spasm before admission aa8 ab8 admission ab8 ac8 after admission ac8

aa9 tremble of hand and foot before admission aa9 ab9 admission ab9 ac9 after admission ac9

aa10 limb weakness before admission aa10 ab10 admission ab10 ac10 after admission ac10

aa11 paralysis of limbs before admission aa11 ab11 admission ab11 ac11 after admission ac11

aa12 neck stiffness before admission aa12 ab12 admission ab12 ac12 after admission ac12

aa13 nuchal rigidity before admission aa13 ab13 admission ab13 ac13 after admission ac13

aa14 Kerning sig before admission aa14 ab14 admission ab14 ac14 after admission ac14

aa151 tendon hyporeflexia before admission aa151 ab151 admission ab151 ac1 5after admission ac15 (0 no 1 unilateral 2 bilateral)

aa161 tendon areflexia before admission aa161 ab161 admission ab161 ac1 6after admission ac16 (0 no 1 unilateral 2 bilateral)

aa17 somnolence before admission aa17 ab17 admission ab17 ac17 after admission ac17

aa18 lethargy before admission aa18 ab18 admission ab18 ac18 after admission ac18

aa19 light coma before admission aa19 ab19 admission ab19 ac19 after admission ac19

aa20 deep coma before admission aa20 ab20 admission ab20 ac20 after admission ac20

aa21 pupil state before admission aa21 ab1 admission ab21 ac21 after admission ac21 (equal and round miosis mydriasis)

aa22 pupillary light reflex before admission aa22 ab22 admission ab22 ac22 after admission ac22 (0 normal 1 abnormal)

**Respiratory system**

ba1 cough before admission ba1 bb1 admission bb1 bc1 after admission bc1

ba2 Pharyngodynia before admission ba2 bb2 admission bb2 bc2 after admission bc2

ba3 nasal obstruction before admission ba3 bb3 admission bb3 bc3 after admission bc3

ba4 running nose before admission ba4 bb4 admission bb4 bc4 after admission bc4

ba5 tachypnea(breathlessness) before admission ba5 bb5 admission bb5 bc5 after admission bc5

ba6 dyspnea before admission ba6 bb6 admission bb6 bc6 after admission bc6

ba7 breathing slows before admission ba7 bb7 admission bb7 bc7after admission bc7

ba8 respiratory rhythm changed before admission ba8 bb8 admission bb8 bc8 after admission bc8

ba9 oral cyanosis before admission ba9 bb9 admission bb9 bc9 after admission bc9

ba101 foam phlegm (sputum) before admission ba101 bb101 admission bb101 bc10 after admission bc10 (sputum color: 0 colorless 1 white 2 pink 3 blood color)

ba11 lung phlegm song before admission ba11 bb11 admission ab11 bc11 after admission bc11

ba12 lung moist rale before admission ba12 bb12 admission bb12 bc12 after admission bc12

**Circulatory system**

ca1 abnormal skin color before admission ca1 cb1 admission cb1 cc1 after admission cc1

ca2 finger, toe or oral cyanosis before admission ca2 cb2 admission cb2 cc2 after admission cc2

ca3 facial and hand-foot-end pale grey before admission ca3 cb3 admission cb3 cc3 after admission cc3

ca4 the whole body cyanosis, pale, grey before admission ca4 cb4 admission cb4 bc4 after admission cc4

ca5 skin pattern before admission ca5 cb5 admission cb5 cc5 after admission cc5

ca6 heart rate increased(more than 120) before admission ca6 cb6 admission cb6 cc6 after admission cc6

ca7 heart rhythm changed(arrhythmia) before admission ca7 cb7 admission cb7 bc7after admission bc7

ca8 pulse turned light and rapid before admission ca8 cb8 admission cb8 cc8 after admission cc8

ca9 pulse weakened before admission ca9 cb9 admission cb9 cc9 after admission cc9

ca10 limb coldness before admission ca10 cb10 admission cb10 bc10 after admission cc10

**Digestive System**

da1 vomit before admission da1 db1 admission db1 dc1 after admission dc1

da2 brown vomitus before admission da2 db2 admission db2 dc2 after admission dc2

da3 abdominal distention before admission da3 db3 admission db3 dc3 after admission dc3

da4 diarrhea before admission da4 db4 admission db4 dc4 after admission dc4

da5 haematemesis before admission da5 db5 admission db5 dc5 after admission dc5

da6 hematochezia before admission da6 db6 admission db6 dc6 after admission dc6

**Date of investigation:**
